# Characterization of Molecular Species and Anti-Inflammatory Activity of Purified Phospholipids from Antarctic Krill Oil

**DOI:** 10.3390/md19030124

**Published:** 2021-02-25

**Authors:** Li Zhou, Xing Wu, Fu Yang, Minghao Zhang, Rong Huang, Jikai Liu

**Affiliations:** 1 School of Pharmaceutical Sciences, South-Central University for Nationalities, Wuhan 430074, China; zhou2018@scuec.edu.cn (L.Z.); wuxing1996@hotmail.com (X.W.); yangfu19951112@icloud.com (F.Y.); minghaozhang@scuec.edu.cn (M.Z.); 2 National Demonstration Center for Experimental Ethnopharmacology Education, South-Central University for Nationalities, Wuhan 430074, China

**Keywords:** Antarctic krill oil, phospholipids, UHPLC-Q-TOF-MS, anti-inflammation

## Abstract

The phospholipids (PLs) from Antarctic krill oil were purified (>97.2%) using adsorption column chromatography. Forty-nine PL molecular species were characterized by ultrahigh-performance liquid chromatography-quadrupole-time-of-flight mass spectrometry (UHPLC-Q-TOF-MS). Most of molecular species contained eicosapentaenoic acid (EPA, 20:5), docosahexaenoic acid (DHA, 22:6), docosapentaenoic acid (DPA, 22:5), and arachidonic acid (AA, 20:4). Notably, a special species PC (20:5/22:6) (1298.17 nmol/g) and many ether PLs were detected. The Antarctic krill PL liposome (IC_50_ = 0.108 mg/mL) showed better anti-inflammatory activity than crude Antarctic krill oil (IC_50_ = 0.446 mg/mL). It could block NF-κB signaling pathway via suppression of IκB-α degradation and p65 activation and dose-dependently reduce the cellular content of inflammatory mediators including nitric oxide (NO), reactive oxygen species (ROS), and inflammatory cytokines in lipopolysaccharide (LPS)-stimulated RAW 264.7 cells. In addition, it can suppress carrageenan-induced mouse paw swelling. Results from the present study could provide a reference for better evaluation of nutritional and medicinal values of Antarctic krill oil.

## 1. Introduction

Inflammatory response is a defense mechanism that evolved in higher organisms to protect them from pathogens and injury, whereby the tissue is repaired, or the pathogenic insult is eliminated. However, when the regulatory mechanisms of the inflammatory response are defective, prolonged and excessive inflammation may give rise to chronic illnesses, for instance, heart disease, arthritis, diabetes, and Alzheimer’s disease [1]. The most known lipid mediators implicated in inflammatory physiological responses are the eicosanoids and platelet-activating factor. Therefore, those related pathways have been considered as promising targets in respect to dietary interventions, especially foods containing bioactive phospholipids (PLs) [2]. To date, there is extensive research published on functional properties of PLs from dietary sources in relation to inflammation-related disorders, which attracted a close attention to their molecular structures and reputed health benefits [3,4]. Marine PLs could reduce inflammation by producing inflammation-resolving mediators from intramolecular eicosapentaenoic acid (EPA) and docosahexaenoic acid (DHA) [5]. Mechanisms underlying the anti-inflammatory activity of marine PLs polyunsaturated fatty acids (PUFAs) include altered cell-membrane PL fatty-acid composition, inhibition of activation of the proinflammatory transcription factor, and activation of peroxisome proliferator activated receptor γ and binding to the G-protein-coupled receptor GPR120 [6].

Antarctic krill (*Euphausia superba*), one of a largest of 86 krill species, inhabits the Southern Ocean. It is a rich source of long-chain omega-3 polyunsaturated fatty acids (n-3 PUFAs), in particular, EPA and DHA. Furthermore, n-3 PUFAs in Antarctic krill oil are mainly bond to PL rather than triglyceride (TAG) as compared to fish oil. Previous studies indicated that the n-3 PUFA in PL form is more bioavailable than the TAG form [7]. However, its bioactive function as a novel food ingredient has not yet been fully explored. Several studies about the anti-inflammatory activities of Antarctic krill oil normally focused on crude krill oil [8,9,10], and little is known about anti-inflammatory property of purified Antarctic krill PL. Poor sample preparation and lipid characterization using mixtures of neutral and polar lipids cannot link the relevant bioactivities to specific lipid classes. Thus, we aim to investigate the anti-inflammatory activity and its underlying mechanism of purified Antarctic krill PL through in vitro and in vivo experiments.

With the development of lipidomics, mass spectrometry (MS) has been widely used in lipid-structures analysis. Time-of-flight mass spectrometry (TOF-MS) is a successful technique for the characterization of molecular structure of lipids due to its precise mass accuracy, high resolution, and excellent sensitivity. Several studies reported the PL profiles in Antarctic krill oil; however, most of them only described the relative abundance of Antarctic krill PL [11,12]. Our present study exhibits for a detail characterization of molecular structures of Antarctic krill PL using ultrahigh-performance liquid chromatography-quadrupole-time-of-flight mass spectrometry (UHPLC-Q-TOF-MS) analysis.

In this study, the PLs from Antarctic krill oil were purified by adsorption column chromatography and subsequently characterized by UHPLC-Q-TOF-MS. In addition, the anti-inflammatory activity with a potential mechanism of purified Antarctic krill PL was comprehensively evaluated.

## 2. Results and Discussion

### 2.1. Separation of Antarctic Krill PL by UHPLC-Q-TOF-MS

**Figure 1:**
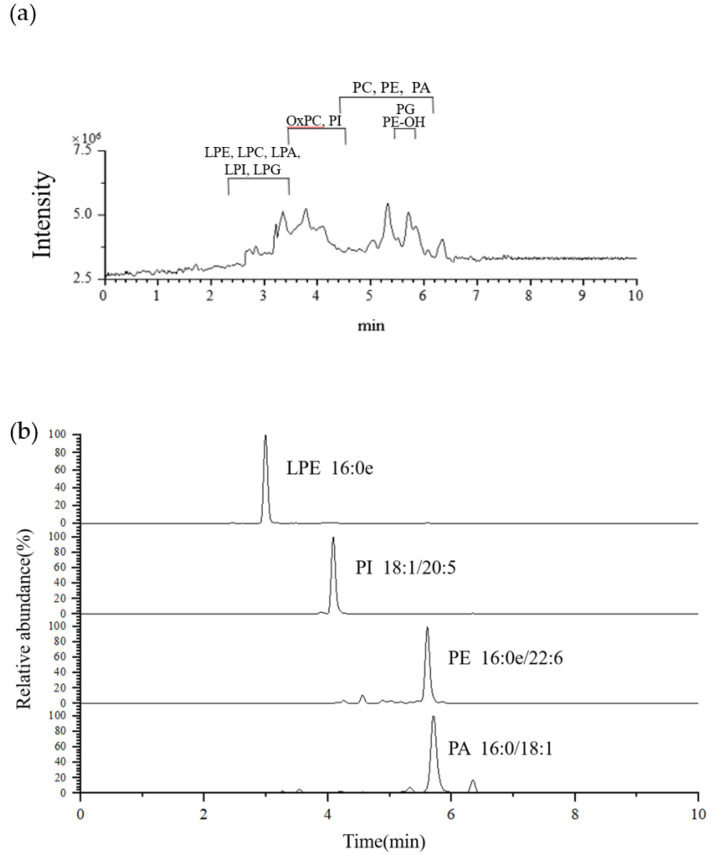
(**a**) Total ion chromatogram of molecular species of Antarctic krill phospholipids (PL) under negative ion mode. (**b**) Extracted individual ion chromatograms obtained from Antarctic krill PL showing the species LPE (16:0e), PI (18:1/20:5), PE (16:0e/22:6), and PA (16:0/18:1).

The Antarctic krill PL was purified from crude oil by column chromatography, and the purity (>97.2%) was checked by HPLC-ELSD. All the characteristics fragments required for the structural elucidation of Antarctic krill PL can be collected under negative electrospray ionization mode. Due to the difference of polarities and the electric charges of PL classes, PE, LPE, PE-OH, PG, LPG, PI, LPI, and PA were detected as [M − H]^−^ ions, PC, LPC, and OxPC were detected as [M + CH_3_COO]^−^ ions. Figure 1a shows the total ion chromatograms of Antarctic krill PLs under ESI source in negative ion mode. A representative extracted individual ion chromatogram (EIC) of *m/z* 438.2992, 881.5224, 748.5334, and 673.4848 was given in Figure 1b. 

The calibration curves for the quantification of PL molecular species presented linear regression coefficients (R2) higher than 0.99, and LOD was ≤0.6 ng/mL (Table S2). The five standards were spiked into the Antarctic krill PLs as reference standards. The recoveries of these standards were between 90 and 110%.

### 2.2. Identification and Quantification of PL Molecular Species 

Table 1 showed the identification and quantification of Antarctic krill PL molecular species. Eleven types of PL classes were detected, and within the PLs, 49 PLs were characterized using UHPLC-Q-TOF-MS/MS. PE and PC were the major PL classes which were in high level. Some other lysophospholipids, such as LPE, LPC, LPG, and LPI, were also detected. These lysophospholipids might be attributed to the hydrolysis of PL caused during storage or pretreatment of krill material [13]. They play important roles in the structure of cellular membranes and participate in the signaling process [14]. In addition, many types of ether phospholipids were distributed in PC, LPE, and PE, whose content is 704.1 nmol/g, accounting for 6.10% of the total content of krill phospholipids (11544.2 nmol/g). Ether phospholipids are peroxisome derived glycerophospholipids in which the hydrocarbon chain at the sn-1 position of the glycerol backbone is attached by an ether bond, as opposed to an ester bond in the more common diacyl phospholipids [15]. This seemingly simple biochemical change has profound structural and functional implications in cell differentiation and signaling pathways. Ether phospholipids constitute approximately 20% of the total phospholipid pool in immune cell membranes; thus, krill oil ether phospholipids could modify the ether lipidome of immune cells, which is important in regulating production of lipid mediators in response to an inflammatory stimulus [16,17,18]. Several previous literatures reported the PL composition of krill oil, but the PL molecular species and concentration exhibited certain differences. For instance, some reasonable amounts of alkenyl phospholipids were detected in the literature of Sung et al. [19]. Furthermore, Song et al. [20] detected several PI species, which were not often reported in lipidomic analysis of krill oil. Those molecular species were not detected in our study. Thus, the phenomenon might be related to the growing environment of Antarctic krill, the extraction technology, or identification methodology.

**Figure 2:**
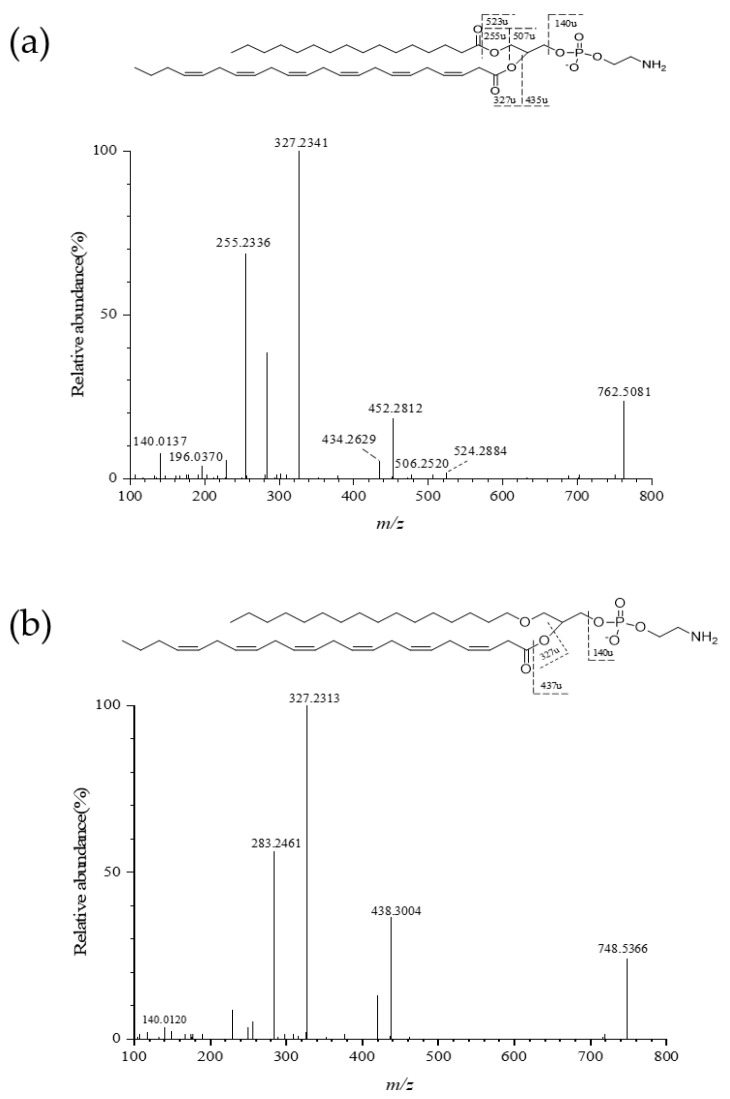
MS/MS fragmentation pathway of (**a**) PE (16:0/22:6) (*m/z* 762.5118) and (**b**) PE (16:0e/22:6) (*m/z* 748.5334) under negative ion mode.

A representative MS/MS fragmentation spectrum of PE (16:0/22:6) and PE (16:0e/22:6) was selected to describe the fragmentation pattern (Figure 2). PE (16:0/22:6) was the most abundant molecular species in krill PE (164.65 nmol/g of purified Antarctic krill PLs, 53.8% of total PE) (Table 1). The result was in agreement with our previous study, which showed that PE (16:0/22:6) and PE (16:0e/22:6) were the major species in purified PE fractions in krill oil [21]. *m/z* 140.0137 corresponded to the characteristic fragment of ethanolamine group. The neutral loss of 255.2336 and 327.2341 between MS and MS^2^ could be attributed to the palmitic acid and decosahexaenoic acid, respectively. *m/z* 506.2520 and 434.2629 corresponded to the loss of docosahexaenoyl acyl group and palmitoyl acyl group, respectively. The detected ion at *m/z* 524.2884 arises from losing moiety of palmitic acid of PE molecular species (Figure 2a). PE (16:0e/22:6) was ionized to generate the precursor ion [M − H]^−^ (*m/z* 748.5334) in the ESI- mode (Figure 2b). *m/z* 327.2313 represented fatty-acid (C22:6) fragment. *m/z* 438.3004 resulted from the loss of linoleic acyl group. The MS/MS fragmentation spectrums of several other molecular species of different PL class were given in Figure S1. The identification performed in this study did not take into account the position of fatty-acyl group in the glycerol moiety.

As shown in Table 1, most of the molecular species contained PUFAs. It was noteworthy that the long chain PUFAs, such as DHA (22:6), EPA (20:5), and DPA (22:5) were widely distributed in Antarctic krill PL. Although the amount of (16:0–20:4) PC (2988.04 nmol/g) showed high level, arachidonic acid (AA) (20:4) was considered to be proinflammatory substrate, the total content of n-3 PUFAs-PL (EPA-PL, DHA-PL, and DPA-PL) was much more abundant than AA, which play a vital role in regulation of inflammation. Those n-3 PUFA could be also able to mediate anti-inflammatory effect through its metabolites such as resolvins, protectins, and insulin sensitizing effect [22]. The above claim was further confirmed by the subsequent evaluations of the anti-inflammatory effects of Antarctic krill PL.

### 2.3. Physicochemical Properties of Liposomes

Figure S3a shows a typical size distribution profile of the liposome with a mean diameter of 188.4 nm. The PDI value of Antarctic krill PL liposome was 0.147, which indicated that the liposomes had a narrow particle-size distribution. Particle charge is an indicator for the characterization of the stability of aqueous nanosuspensions. The zeta potential of the Antarctic krill PL liposome was −43.6 mV (Figure S3b). It indicated that the Antarctic krill PL liposome is stable, because a minimum of ± 30 mV is required for a physical stable nanosuspension solely stabilized by electrostatic repulsion [23].

### 2.4. Effect of Antarctic Krill PL Liposome on Cell Viability, Nitric Oxide (NO) Production, and Proinflammatory Cytokines Secretion in Lipopolysaccharide (LPS)-stimulated RAW 264.7 Cells

**Figure 3:**
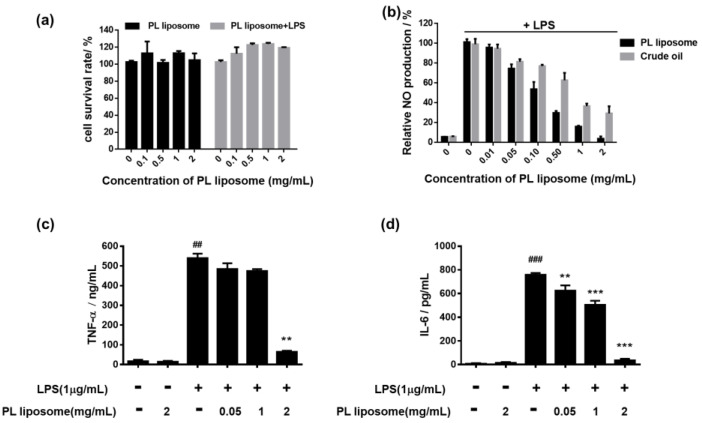
(**a**) Effect of Antarctic krill PL liposome on RAW 264.7 cells viability. (**b**) Effect on inflammatory mediator NO production compared with crude Antarctic krill oil in lipopolysaccharide (LPS)-induced RAW 264.7 cells by Griess assay. (**c**,**d**) Effects on the secretion of proinflammatory cytokines (**c**) TNF-α and (**d**) IL-6 by ELISA. Data are expressed as mean ± S.D of three independent experiments. ** *p <* 0.01 and *** *p <* 0.001 compared with LPS-treated group. ^##^ *p <* 0.01 and ^###^ *p <* 0.001 compared with control group.

LPS-stimulated RAW 264.7 cells are commonly used for screening anti-inflammatory drug candidates. First of all, the cytotoxic effects of Antarctic krill PL liposome on macrophage RAW 264.7 cells were examined by MTT assay (Figure 3a). The cells were incubated with PL liposome at various concentrations (0, 0.1, 0.5, 1, and 2 mg/mL) for 1 h without or with LPS stimulation for a further 24 h. Cell viability in each group was 95–120%, indicating no cytotoxicity of our Antarctic krill PL liposome on both normal and LPS-induced RAW 264.7 cells.

NO interacts with a series of cellular signaling pathways and plays a critical role in various forms of inflammation and carcinogenesis. The effect of Antarctic krill PL liposome and crude oil on NO secretion in cell supernatants was evaluated using Griess reagent as shown in Figure 3b. Compared to the control group, NO level was remarkably increased after stimulated with LPS for 18 h, which means the model of LPS-induced inflammation was successfully established. However, pretreatment with PL liposome significantly suppressed NO production in a dose-dependent manner with IC_50_ = 0.108 mg/mL. Meanwhile, crude Antarctic krill oil showed only moderate NO scavenging effects with IC_50_ = 0.446 mg/mL. These results indicated that purified Antarctic krill PL liposome might serve as a better anti-inflammatory agent with higher bioavailability than crude oil.

### 2.5. Effects of Antarctic Krill PL Liposome on the Secretion of Proinflammatory Cytokines

To further evaluate the impacts of Antarctic krill PL liposome on proinflammatory cytokine levels, we collected cell supernatants and performed ELISA. In Figure 3c,d, LPS-stimulation enhanced the concentrations of TNF-α and IL-6 by nearly 50-fold. Meanwhile, Antarctic krill PL liposome treatment with concentrations of 0.05, 1, and 2 mg/mL showed a significant reduction by 17.6, 19.3, and 87.2% in TNF-α level and 23.6, 33.1, and 94.0% in IL-6 level, respectively. These data are consistent with the results of NO production, suggesting that Antarctic krill PL liposome can regulate the immune response and the release of inflammatory mediators.

Under the stimulus of pathogens, activated macrophages secrete proinflammatory cytokines to elicit an inflammatory response [24]. TNF-α is an essential cytokine in the inflammatory cytokine network, which can trigger endotheliocytes and leukocytes to release various inflammatory mediators accompanied by fever symptoms [25]. IL-6 is a multifunctional cytokine with both pro- and anti-inflammatory properties in the immune response, such as rheumatoid arthritis [26]. Therefore, down-regulation of these inflammatory cytokines is of utmost importance during anti-inflammatory therapy.

Both n-3 and n-6 PUFAs act as precursors for the synthesis of various eicosanoids and docosanoids, which are related to protective effects on inflammation diseases. When an external signal stimulates the immune cell membrane, cytosolic phospholipase A2 is activated to release free fatty acid from the membrane PLs [27]. The free AA (arachidonic acid) then acts as a substrate for cyclooxygenase (COX), lipoxygenase (LOX), or cytochrome P450 enzymes, which can metabolize into several lipid mediators [28]. DHA and EPA has been shown to inhibit arachidonic acid metabolism and to decrease expression of the COX-2 gene [29]. The engulfed krill PL liposome may affect the composition of n-3/n-6 PUFAs in the PL membrane of macrophages, which seem to be important in regulating production of lipid mediators in response to an inflammatory stimulus.

### 2.6. Effect of Antarctic Krill PL Liposome on Intracellular Reactive Oxygen Species (ROS) Level

The effects of krill PL liposome on oxidative stress were assessed by an activatable-fluorescent DCFH-DA probe. In Figure S2, the results showed that LPS stimulation clearly elevated the cellular ROS levels, while this promotion was significantly suppressed by cotreatment with krill PL liposome at 1 and 2 mg/mL (*p* < 0.001).

As an important marker of in vitro macrophage activation, overproduction of ROS stimulated by self-cellular oxidative stress results in NF-κB activation and downstream expression of iNOS, COX-2, IL-6, and TNF-α. Richard et al. found supplemented cells with n-3 PUFAs produced lower amounts of ROS than cells fed with n-6 PUFAs [30]. Chakraborty et al. reported that n-3 PUFAs supplements can reduce ROS level in LPS-stimulated macrophages [31]. Antarctic krill PL liposome with high n-3/n-6 ratio might activate the endogenous antioxidative mechanisms to synthetically protect from inflammatory response. In addition, trivial residuals including vitamin A, vitamin E, and astaxanthin in Antarctic krill PL are likely resistant to oxidation [32].

### 2.7. Effects of Antarctic Krill PL Liposome on Protein and mRNA Expressions of iNOS and COX-2 in LPS-Induced RAW 264.7 Cells

**Figure 4:**
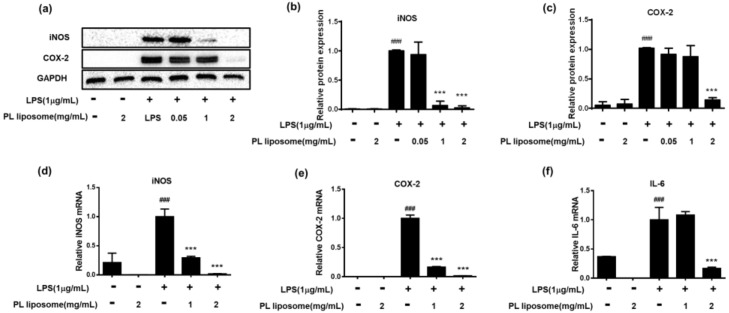
(**a**–**c**) Effect of Antarctic krill PL liposome on protein expressions of iNOS and COX-2 in RAW 264.7 cells. (**a**) Protein expression measured by western blot assay; gray density scanning analysis of iNOS (**b**) and COX-2 (**c**) normalized to GAPDH. (**d**–**f**) Effect of Antarctic krill PL liposome on mRNA levels of iNOS (**d**), COX-2 (**e**), and IL-6 (**f**) in RAW 264.7 cells. GAPDH was used as internal reference. Data are expressed as mean ± S.D of triplicates. *** *p <* 0.001 compared with LPS-treated group. ^###^ *p <* 0.001 compared with control group.

Antarctic krill PL liposome was also examined whether it can reduce protein and mRNA expression of inflammation-associated molecules (Figure 4). Through western blot analysis (Figure 4a–c), it suggested that 1 μg/mL of LPS can successfully induce protein expression of iNOS and COX-2 (*p* < 0.001). However, pretreatment of krill PL liposome at the dose of 2 mg/mL can significantly down-regulate these protein expressions as compared to LPS alone-treated group (*p* < 0.001). Future studies should seek to investigate mRNA expression of inflammatory factors upon PL liposome treatment through qRT-PCR (Figure 4d–f). Consistent with the above findings, after cotreatment with LPS and PL liposome for 18 h, mRNA levels of iNOS, COX-2, and IL-6 were significantly inhibited. Therefore, Antarctic krill PL liposome can suppress both transcriptional and translational levels of inflammatory mediators in the macrophages.

iNOS and COX-2 are considered to be important proinflammatory mediators, and their expressions are associated with the transcriptional activation. Up-regulation of iNOS and subsequent production of NO are very crucial in macrophages toward infectious agents [33]. The molecular target of nonsteroidal anti-inflammatory drugs (NSAIDs), inducible COX-2, is closely involved in the formation of inflammatory prostaglandins (PG) from arachidonic acid. Various natural PLs from plant, animal, and marine origin have been shown anti-inflammatory activities through suppression of iNOS and COX-2 [34].

### 2.8. Effects of Antarctic Krill PL Liposome on NF-κB Subunits Activation in LPS-Stimulated RAW 264.7 Cells

**Figure 5:**
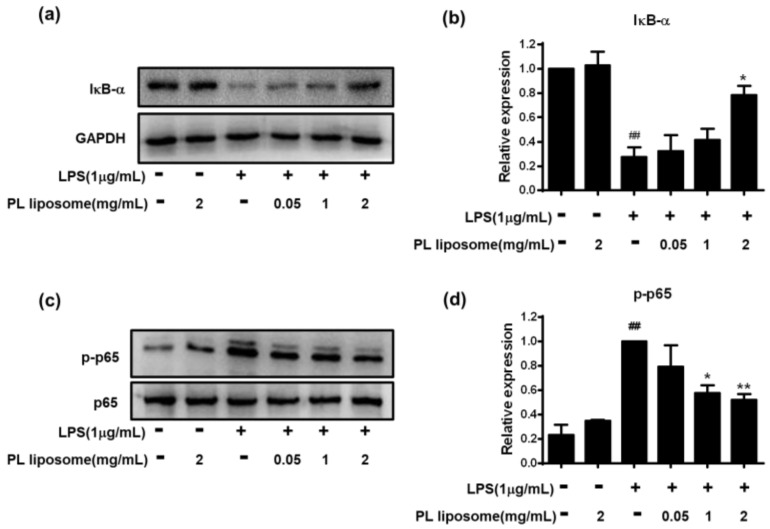
Effects of Antarctic krill PL liposome on NF-κB subunits activation in LPS-stimulated RAW 264.7 cells. (**a**,**b**) IκB-α normalized to GAPDH; (**c**,**d**) p-p65 normalized to p65. Data are represented as mean ± S.D of three independent experiments. * *p <* 0.05 and ** *p <* 0.01 compared with LPS-treated group. ^##^ *p <* 0.01 compared with control group.

To further reveal the underlying mechanism involved in this anti-inflammatory effect, NF-κB signaling pathways were observed. Cells were incubated with different concentration of PL liposome for 1 h, followed by treatment with LPS (1 μg/mL) for 6 h. Cell lysates were extracted, and the protein levels of IκB-α and p-p65 were analyzed by western blot (Figure 5). Our results showed that LPS strongly induced IκB-α decrease and p65 phosphorylation (*p <* 0.01), while preincubation with PL liposome at the dose of 2 mg/mL blocked those changes obviously (*p <* 0.05 for IκB-α and *p <* 0.01 for p-p65). Taken together, these observations demonstrated that Antarctic krill PL liposome can interfere with the NF-κB signaling pathway via suppression of IκB-α degradation and p65 activation, which contribute to the down-regulation of downstream associated genes including those of iNOS, COX-2, and inflammatory cytokines.

NF-κB is a ubiquitous, inducible transcription factor responsible for mediating the expression of many genes involved in inflammation, oxidative stress, injury, apoptosis, and proliferation [35]. NF-κB is a complex of dimeric subunits that belong to the NF-κB/Rel family. As the main functional element, p65 was involved in transcriptional regulation of various physiological and pathological events, meanwhile IκB has been identified as an NF-κB p65-binding protein, which prevents p65 phosphorylation and translocation to the nucleus, thereby decreasing the production of cytokines [36]. To our knowledge, n-3 PUFAs can modulate the expression of several inflammatory genes by reducing NF-κB activity, which subsequently lowers the inflammation and oxidative stress in cells [37].

### 2.9. Effect of Krill PL Liposome on Carrageenan-Induced Mouse Paw Edema

**Figure 6:**
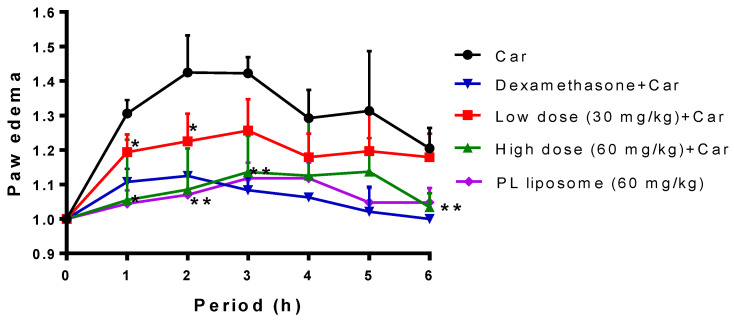
The inhibitory effect of Antarctic krill PL liposome on carrageenan-induced mouse paw edema. Mice were randomly divided into five groups: carrageenan (Car), dexamethasone (a positive control), low dose of krill PL liposome, high dose of PL liposome, and PL liposome control. Paw edema was measured before administration and every hour after carrageenan injection until 6 h. The data represent mean ± SD (*n* = 6), * *p* < 0.05 and ** *p* < 0.01 represent significant difference for low/high dose of PL liposome group compared with the carrageenan-treated group.

The carrageenan-induced mouse-paw-edema model was used to evaluate the anti-inflammatory effect of Antarctic krill PL liposome in vivo. As in shown in Figure 6, injection of carrageenan stimulated local inflammation and then caused edema in the paw tissue. The mean paw thickness ratio (right paw/left paw) in the carrageenan group peaked at approximately 1.5 after 2 h, and paw edema was persisting up to 3 h, followed by a gradual decline. Dexamethasone was used as a positive control, indicating that 3 mg/kg of dexamethasone pretreated for 30 min then stimulated with carrageenan could inhibit paw edema. Similarly, pretreatment with 30 or 60 mg/kg Antarctic krill PL liposome could markedly attenuate paw edema during the first 3 h after carrageenan injection (*p <* 0.05 or *p <* 0.01), which reveals that Antarctic krill PL liposome accelerates the resolution of paw edema due to its favorable anti-inflammatory effects.

As an extensively used model for selecting candidates to relieve acute inflammation, injection of carrageenan dramatically increases the levels of TNF-α, IL-1β, NO, and PGE2 in the swelling paw [21]. Kao et al. showed that that an administration of squid-skin liposomes dose-dependently decreased carrageenan-induced swelling [38]. Hence, Antarctic krill PL liposome is a combination of major anti-inflammatory fatty acids, which might be responsible for its significant activity in the in vitro and in vivo inflammatory model.

## 3. Materials and Methods

### 3.1. Chemicals

Antarctic krill oil were provided by Liaoning Province Dalian Ocean Fishery Group of Corporations (Dalian, China). PL standards (15:0–18:1-d7-PC, 15:0–18:1-d7-PE, 15:0–18:1-d7-PG, 15:0–18:1-d7-PI, and 15:0–18:1-d7-PA) were purchased from Avanti Polar Lipids (Alabaster, AL, USA). Chloroform (CHCl_3_), methanol (MeOH), acetonitrile (ACN) and isopropanol (IPA), formic acid, and ammonium formate were purchased from Macklin Biochemical Technology Co., Ltd. (Shanghai, China).

Primary antibodies against iNOS, COX-2 were purchased from Cell Signaling Technology (Boston, MA, USA). The ELISA kits of IL-6 and TNF-α were purchased from Nanjing Jiancheng Bioengineering Institute (Nanjing, China). SYBR Green Master Mix for qPCR was purchased from Thermo Fisher Scientific (Shanghai, China). All other reagents were commercially available and of analytical grade.

### 3.2. Antarctic Krill PL Purification

Antarctic krill oil was dissolved in CHCl_3_ and then transferred onto a silica gel column preconditioned with n-heptane. Elution was performed using CHCl_3_: MeOH (88:12, *v/v*). Lipids in elution fractions were monitored by thin-layer chromatography with silica gel 60 F254 (Merck, Darmstardt, Germany), CHCl_3_-CH_3_OH25% aqueous ammonia 60:35:5 (*v/v*) as mobile phase and phosphomolybdic acid in ethanol (20:80, *v/v*) as chromogenic agent. PL classes were identified in comparison of the retention factor (Rf) to PL standards as references. Fractions containing pure PLs were collected and evaporated. The purity of Antarctic krill PL was tested according to a previous study [39]. The final purified PL sample was re-dissolved in IPA prior to UHPLC-Q-TOF-MS analysis.

### 3.3. UHPLC-Q-TOF-MS Analysis

Antarctic krill PL was analyzed using a Shimadzu LC-30A system coupled to a Triple-TOF 6600 mass spectrometry with a 2.6 μm, 100 mm × 2.1 mm Kinetex C18 column (Phenomenex, Torrance, CA, USA). H_2_O/MeOH/ACN (1:1:1, *v/v/v*) and IPA/ACN (5:1, *v/v*) were used as mobile phase A and B, respectively, and both mobile phases contained 5 mM ammonium acetate. The binary gradient for PL separation was as follows: 0–0.5 min, 80% A, 0.5–1.5 min, 80–60% A, 1.5–3 min, 60–40% A, 3–8 min, 40–2% A, 8–10 min, and 2–80% A. The flow rate was set at 0.4 mL/min. The column temperature was set at 60 °C. After each analysis, the column was flushed with 80% of the mobile phase A for 5 min before the beginning of the next analysis. Data were collected in negative ionization mode. The collision energy of 30 eV was used for PA, 35 eV for other PLs.

### 3.4. Characterization of PL Molecular Species

Five deuterium (d7)-labeled PLs as standards, including 15:0–18:1-d7-PC (standard for PC, lysophosphatidylcholine (LPC)), 15:0–18:1-d7-PE (standard for PE, lysophosphatidylethanolamine (LPE), ether PE and phosphatidylethanol (PE-OH)), 15:0–18:1-d7-PG (standard for PG, lysophosphatidylglycerol (LPG)), 15:0–18:1-d7-PI (standard for PI, lysophosphatidylinositol (LPI)), and 15:0–18:1-d7-PA (standard for PA). A quality control (QC) sample (cocktail of each sample) were injected regularly every six samples.

The five PL standards were added in Antarctic krill PL and QC samples. Peak areas of the PL standards and Antarctic krill PL were integrated from EICs. The concentration of PLs was determined on the basis of different standard calibration curves.

### 3.5. Preparation and Characterization of Antarctic Krill PL Liposome

The Antarctic krill PL liposome was freshly prepared by homogenizing 10 mg Antarctic krill PL and 1 mL serum-free culture medium or PBS using an Ultra-Turrax T18 homogenizer operating at 21,000 g for 3 min, then further homogenized by a high-pressure homogenizer (D-3L, PhD tech., St. Paul, MN, USA) at 700 bar for 2 min. The particle size, polydispersity index (PDI) and zeta potential of liposomes were measured by dynamic light scattering instruments (Zetasizer Nano-ZS, Malvern Instruments, Malvern, UK).

### 3.6. Cell Culture

Murine macrophages RAW 264.7 cells were purchased from China Center for Type Culture Collection (CCTCC, Wuhan, China). Cells were cultured in Dulbecco’s modification of Eagle’s medium (DMEM) supplemented with 10% FBS, 100 U/mL penicillin and 100 μg/mL streptomycin in a humidified atmosphere of 5% CO_2_ at 37 °C in a CO_2_ incubator (Thermo Fisher Scientific, Waltham, MA, USA).

### 3.7. Cell Viability Assay

RAW 264.7 cells were seeded in a tissue culture grade 96-well plates at a density of 5 × 10^4^ cells/well and incubated for 24 h. The cells were treated with Antarctic krill PL liposome at the concentrations of 0, 0.1, 0.5, 1, and 2 mg/mL for 1 h and subsequently stimulated with or without LPS (1 μg/mL) for 24 h. After specific treatment in each group, cells were washed by PBS, incubated with 0.5 mg/mL MTT for 4 h, and then the medium was removed. Formazan crystals produced in each well were dissolved in DMSO (150 μL), and the absorbance was recorded at 568 nm by a microplate reader (Tecan Spark 10M, Männedorf, Switzerland). Cell viability was calculated using the following formula: % Cell viability = (A1/A0) × 100%, where A1 and A0 are the absorbance of the test samples and control, respectively.

### 3.8. Measurement of NO Production

NO production was analyzed through a Griess reagent. RAW 264.7 cells were plated in 96-well at 5 × 10^4^ cells/well, treated with Antarctic krill PL liposome or crude oil (0, 0.01, 0.05, 0.1, 0.5, 1 and 2 mg/mL, respectively) for 1 h followed by stimulating with or without LPS (1 μg/mL) for 18 h. Then, 50 μL supernatants were collected and mixed with equal amount of Griess reagents for 10 min at room temperature. The absorbance of each well was determined at 540 nm by using a microplate reader. Relative NO production was shown as the percentage of release amount in cells treated only with LPS.

### 3.9. Measurement of Intracellular ROS Level

RAW 264.7 cells were plated in black 96-well at 5 × 10^4^ cells/well, treated with Antarctic krill PL liposome (0, 0.05, 1, and 2 mg/mL, respectively) for 1 h followed by stimulate with LPS (1 μg/mL). After 18 h, the cell-culture supernatant was removed, and 50 μL of DCFH-DA probe was added with a final concentration of 10 μM. The absorbance was recorded at 525 nm by using a microplate reader.

### 3.10. Cytokines Quantification by ELISA

The production of TNF-α and IL-6 was measured by enzyme-linked immunosorbent assay (ELISA) according to manufacturer’s protocol. Briefly, RAW 264.7 cells were treated with Antarctic krill PL liposome as described above, then the supernatants were collected and added into ELISA plate for 2 h incubation. After strict wash, biotinylated detection antibody was incubated for 2 h, followed by washing and the addition of streptavidin–horseradish peroxidase (Sav-HRP) for 30 min. The plate was washed, and substrate solution was added for 30 min in dark. Finally, the reaction was stopped by 10% (*v/v*) H_2_SO_4_, and the plate was read on a microplate reader at 450 nm. Standard curves were constructed with TNF-α (0–1000 pg/mL) and IL-6 (0–1000 pg/mL).

### 3.11. Western Blot Analysis

RAW 264.7 cells were seeded in 6-well plates at a density of 2 × 10^5^ cells/well, treated with Antarctic krill PL liposome at the concentrations of 0.05, 1, and 2 mg/mL for 1 h, and then stimulated with 1 μg/mL LPS for 6 h (for IκB-α, p65, and p-p65 proteins extraction) and 18 h (for iNOS and COX-2 proteins extraction). Then, the cells were washed twice with cold PBS and collected for total protein extraction. The cells were lysed on ice for 30 min with RIPA buffer containing a protease-inhibitor cocktail, and the protein concentration was determined by BCA Protein Assay Kit (Beyotime, Shanghai, China). For western blot analysis, the same amount of protein was resolved by 10% sodium dodecyl sulfonate polyacrylamide-gel electrophoresis (SDS-PAGE) and electroblotted onto polyvinylidene fluoride (PVDF) membrane. The membranes were sealed with 5% skim milk (*w/v*, dissolved in Tris-buffered Saline Tween-20, TBST) for 1 h and then incubated overnight at 4 °C with corresponding primary antibodies. Subsequently, the membranes were rinsed for three times in TBST and incubated for 1 h at room temperature with specific goat anti-rabbit IgG secondary antibodies (Beyotime, Shenzhen, China). After repeated wash, chemiluminescent signals of the protein bands were detected using ECL solution and acquired by the imaging system (ChemiDoc XRS+, Bio-Rad, Hercules, CA, USA). The band scanning densitometry of each protein was quantified through Image J.

### 3.12. Quantitative RT-PCR

Total RNA was isolated using the Trizol reagent (Invitrogen, Carlsbad, CA, USA). Specific primers purchased from TsingKe (Beijing, China) are used as follows (Table S1): cDNA was synthesized using a PrimeScript reagent kit (Takara Bio, Kusatsu, Japan) and was analyzed by qPCR using Real-Time PCR System (ABI 7500, Thermo Fisher Scientific; Waltham, MA, USA) with SYBR Green PCR Master Mix (Thermo Fisher Scientific; Waltham, MA, USA). After an initial denaturation step (95 °C for 4 min), the PCR reaction consisted of 40 cycles (95 °C, 15 s; 57 °C, 15 s; and 72 °C, 1 min). Gene expression level was calculated using the comparative 2^−ΔΔCt^ method and normalized to that of GAPDH, which was used as an internal control.

### 3.13. Carrageenan-Induced Paw Edema

Male c57BL/6 mice (18–22 g) were purchased from Huazhong Agricultural university (Certificate SCXK2015-0019; Wuhan, China). All mice were maintained under standard specific pathogen-free conditions with a 12 h light/dark cycle for 1 week. Animal use and care were confirmed by the Animal Experimental Ethics Committee of South-Central University for Nationalities (SYXK (Wuhan) 2016-0089, No. 2019-SCUEC-AEC-014). The mice were divided randomly into five groups (*n* = 6) for further experiments: carrageenan, dexamethasone (a positive control, 3 mg/kg), low dose of Antarctic krill PL liposome (30 mg/kg), high dose of Antarctic krill PL liposome (60 mg/kg), and Antarctic krill PL liposome control. Animals were treated with normal saline, dexamethasone, or Antarctic krill PL liposome (30, 60 mg/kg) through intraperitoneal injection, 30 min prior to injection of 10 mg/mL carrageenan (50 μL) in the plantar side of right hind paws of the mice. For Antarctic krill PL liposome control group, mice were treated only with Antarctic krill PL liposome (60 mg/kg). The paw volume was measured at 0, 1, 2, 3, 4, 5, and 6 h intervals after carrageenan injection and using toe volume measuring instrument (Jinan, China). The degree of swelling was evaluated by the ratio A/B, where A and B are the volumes of the right hind paw after and before carrageenan treatment, respectively.

### 3.14. Statistical Analysis

PL compositions of krill oil were identified and quantified with the use of AB SCIEX Analyst 1.6.2 software (Applied biosystems, Foster city, CA, USA) and LipidMaps. The mass accuracy of the Q-TOF MS instrument was set to ± ~2–3 ppm, as stated a total of ± 5–6 ppm for identification. False positives were checked manually according to the MS/MS fraction analysis. The activity test results were presented as the means ± standard deviation (SD) and multiple comparisons were performed using one-way analysis of variance (ANOVA) followed by Tukey’s post hoc test. Statistical significance was set at *p* < 0.05. Statistical analysis was accomplished by SPSS 16.0 software (San Diego, CA, USA).

## 4. Conclusions

A UHPLC-Q-TOF-MS technique was applied to characterize the molecular species of Antarctic krill PL. High levels of PUFAs were distributed in the fatty-acid chains of PL molecules, such as EPA-PL, DHA-PL, and AA-PL. The Antarctic krill PL liposome could effectively suppress LPS-induced production of NO, ROS, and proinflammatory cytokines in RAW 264.7 cells, down-regulate the expression of iNOS, and COX-2 transcriptionally through blocking the activation of NF-κB signaling pathway, and also reduce mouse paw swelling. Our study reveals the favorable anti-inflammatory activity of Antarctic krill PL owing to high levels of n-3 PUFAs in their fatty-acid chains, which opens up new possibilities for the use of Antarctic krill PL not only as a superior nutritional source of n-3 PUFAs but also for use in the pharmaceutical, cosmetic, and functional food industries.

## Figures and Tables

**Table 1:** Molecular species of krill PL determined by (ultrahigh-performance liquid chromatography-quadrupole-time-of-flight mass spectrometry) UHPLC-Q-TOF-MS.

Precursors	Molecular Species	*m/z* (observed)	*m/z* (calculated)	Error (ppm)	Contents (nmol/g)	Total Contents (nmol/g)
PE						
[M − H]^−^	16:0/18:3	712.4955	712.4923	4.49	2.14	306.05
[M − H]^−^	14:0e/22:6	720.5007	720.4974	4.58	8.67	
[M − H]^−^	17:0/18:2	728.5261	728.5230	4.26	2.82	
[M − H]^−^	18:1e/20:5	748.5259	748.5287	−3.74	3.29	
[M − H]^−^	16:1e/22:5	748.5314	748.5287	3.61	3.07	
[M − H]^−^	16:0e/22:6	748.5334	748.5287	6.28	97.40	
[M − H]^−^	16:0e/22:5	750.5471	750.5443	3.73	3.27	
[M − H]^−^	16:1/22:6	760.4916	760.4917	−0.13	3.79	
[M − H]^−^	16:0/22:6	762.5118	762.5079	5.08	164.65	
[M − H]^−^	20:5/20:5	782.4785	782.4761	3.07	12.80	
[M − H]^−^	20:4/22:6	810.5107	810.5074	4.07	4.15	
LPE						
[M − H]^−^	16:1e	436.2834	436.2828	1.38	4.99	122.93
[M − H]^−^	15:0	438.2641	438.2626	3.42	5.04	
[M − H]^−^	16:0e	438.2992	438.2984	1.83	47.26	
[M − H]^−^	16:1	450.2639	450.2626	2.89	2.48	
[M − H]^−^	18:2e	462.2997	462.2990	1.51	3.96	
[M − H]^−^	18:1e	464.3130	464.3141	−2.37	9.88	
[M − H]^−^	18:0e	466.3291	466.3303	−2.57	14.10	
[M − H]^−^	18:2	476.2797	476.2783	2.94	7.25	
[M − H]^−^	20:1	506.3225	506.3247	−4.35	2.65	
[M − H]^−^	22:6	524.2776	524.2783	−1.34	25.32	
PE-OH						
[M − H]^−^	16:0/18:2	699.4998	699.4970	4.00	12.33	12.33
PC						
[M + CH_3_COO]^−^	14:0e/20:5	796.5502	796.5498	0.50	87.89	8676.65
[M + CH_3_COO]^−^	15:1e/20:5	808.5529	808.5498	3.83	71.45	
[M + CH_3_COO]^−^	14:0/20:5	810.5310	810.5291	2.34	250.36	
[M + CH_3_COO]^−^	16:0/18:4	812.5482	812.5447	4.31	117.95	
[M + CH_3_COO]^−^	16:0/18:3	814.5638	814.5604	4.17	399.92	
[M + CH_3_COO]^−^	14:0e/22:6	822.5657	822.5654	0.36	119.04	
[M + CH_3_COO]^−^	16:1e/20:5	822.5660	822.5654	0.73	131.89	
[M +CH_3_COO]^−^	17:0/18:2	830.5957	830.5917	4.82	180.77	
[M + CH_3_COO]^−^	14:0/22:6	836.5480	836.5447	3.94	207.58	
[M + CH_3_COO]^−^	16:0/20:4	840.5812	840.5760	6.19	2988.04	
[M + CH_3_COO]^−^	16:0/22:5	866.5966	866.5917	5.65	1841.90	
[M + CH_3_COO]^−^	18:1/20:1	872.6412	872.6386	2.98	93.12	
[M + CH_3_COO]^−^	18:0/22:6	892.6121	892.6073	5.38	184.81	
[M + CH_3_COO]^−^	18:0/22:5	894.6262	894.6230	3.58	605.82	
[M + CH_3_COO]^−^	20:5/22:6	910.5667	910.5604	6.92	1298.17	
[M + CH_3_COO]^−^	22:6e/22:6	922.5999	922.5967	3.47	97.94	
LPC						
[M + CH_3_COO]^−^	16:0	554.3469	554.3463	1.08	1123.18	2183.34
[M + CH_3_COO]^−^	18:1	580.3623	580.3620	0.52	307.66	
[M + CH_3_COO]^−^	18:0	582.3772	582.3776	−0.69	326.04	
[M + CH_3_COO]^−^	20:5	600.3318	600.3307	1.83	426.46	
PG						
[M − H]^−^	16:0/20:1	775.5499	775.5495	0.52	16.19	16.19
LPG						
[M − H]^−^	16:1	481.2576	481.2572	0.83	4.81	9.82
[M − H]^−^	16:0	483.2707	483.2728	−4.35	5.01	
PI						
[M − H]^−^	18:1/20:5	881.5224	881.5186	4.31	115.77	115.77
LPI						
[M − H]^−^	16:0	571.2910	571.2889	3.68	10.05	10.05
PA						
[M − H]^−^	16:0/18:2	671.4678	671.4658	3.04	40.24	91.07
[M − H]^−^	16:0/18:1	673.4848	673.4814	5.08	50.83	

## Data Availability

Data is contained within the article and Supplementary Material.

## Supplementary Materials

The following are available online at https://www.mdpi.com/1660-3397/19/3/124/s1, Figure S1: MS/MS fragmentation pathway of (a) PE-OH(16:0/18:2) (*m/z* 699.4998), (b) PC(20:5/22:6) (*m/z* 910.5667), (c) PG(16:0/20:1) (*m/z* 775.5499), (d) PI(18:1/20:5) (*m/z* 881.5224), and (e) PA(16:0/18:2) (*m/z* 671.4678) under negative ion mode. Figure S2: Relative intracellular ROS level was measured with a cell-permeable fluorescence-activatable probe DCFHDA. Data are expressed as mean ± S.D of triplicates. *** *p* < 0.001 compared with LPS-treated group. ## *p* < 0.01 compared with control group. Figure S3: Size distribution (a) and zeta potential distribution (b) of krill PL liposomes. Table S1: Primers for qPCR assay. Table S2: Calibration curves, linear regression coefficients (R2), limit of detection (LOD), and limit of quantification (LOQ) of five PL standards.
