# Supplementary material for: Characterization of Molecular Species and Anti-Inflammatory Activity of Purified Phospholipids from Antarctic Krill Oil
Source: Mar Drugs. 2021 Feb 25;19(3):124. doi: 10.3390/md19030124 (PMC7996531; doi:10.3390/md19030124)
Supplement: Supplementary file 1 [file marinedrugs-19-00124-s001.pdf]

(a)

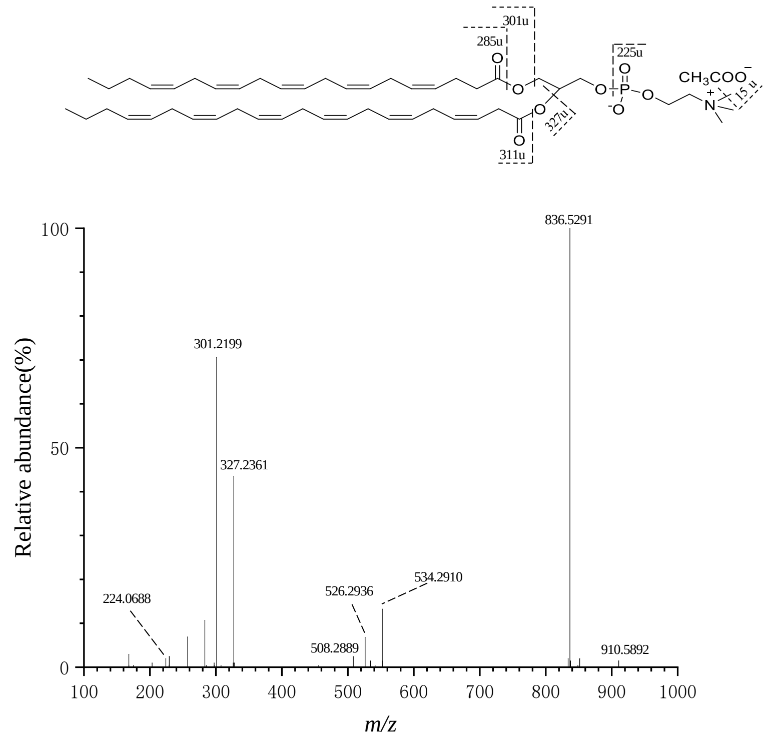
301u
285u
225u
CH_3COO^-
15 u
327u
311u
836.5291
301.2199
327.2361
224.0688
526.2936
534.2910
508.2889
910.5892
Relative abundance(%)
m/z

(b)

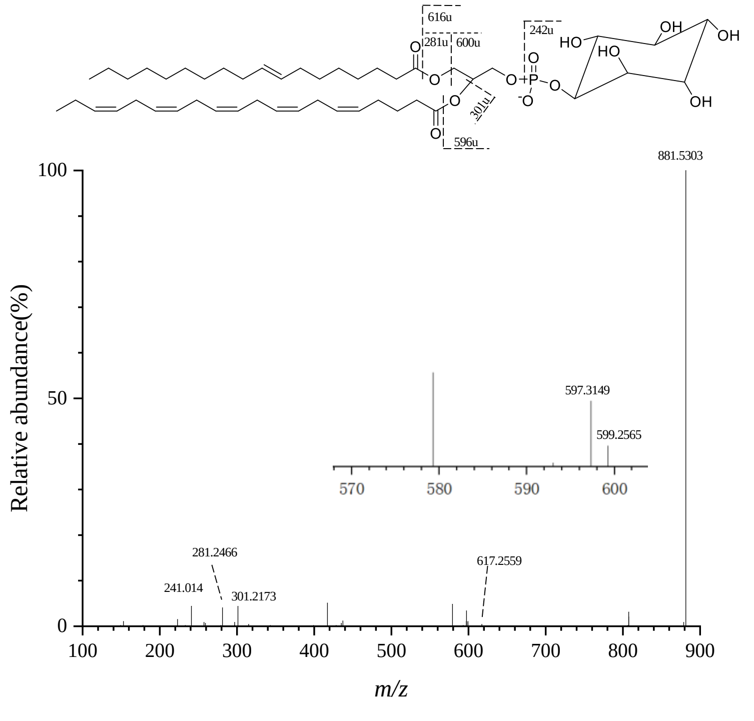
616u
281u
600u
242u
301u
596u
881.5303
597.3149
599.2565
281.2466
241.014
301.2173
617.2559
Relative abundance(%)
m/z

(c)

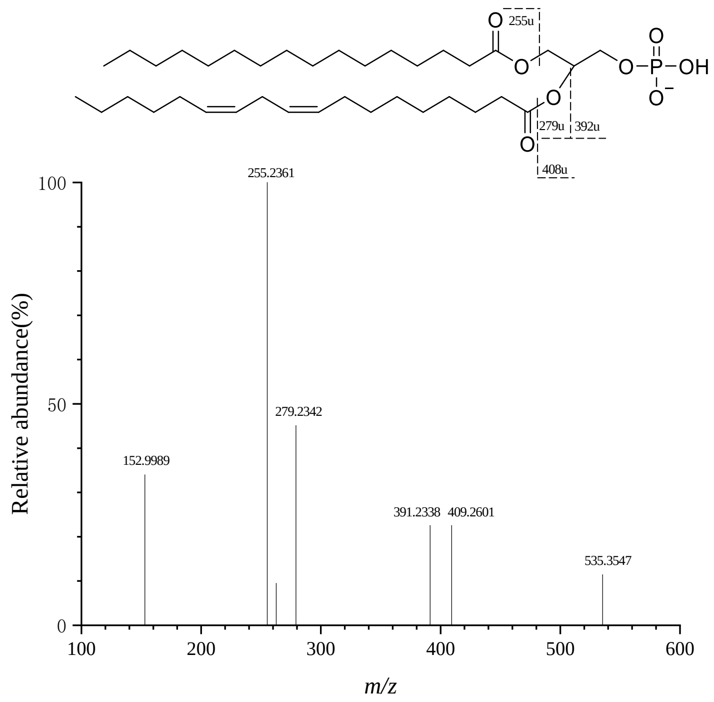

Figure S1. MS/MS fragmentation pathway of (a) PC(20:5/22:6) (*m/z* 910.5667), (b) PI(18:1/20:5) (*m/z* 881.5224), (c) PA(16:0/18:2) (*m/z* 671.4678) under negative ion mode.

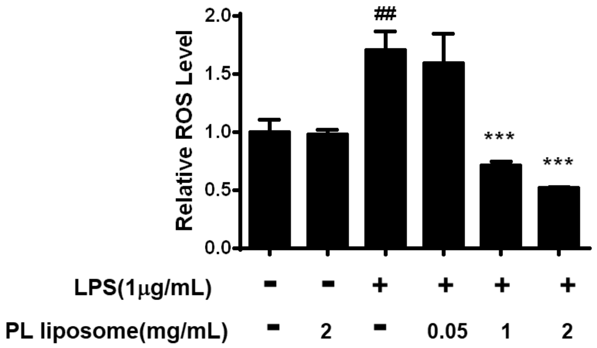

Figure S2. Relative intracellular ROS level was measured with a cell-permeable fluorescence-activatable probe DCFHDA. Data are expressed as mean ± S.D of triplicates. ***p < 0.001 compared with LPS-treated group. ##p < 0.01 compared with control group.

(a)

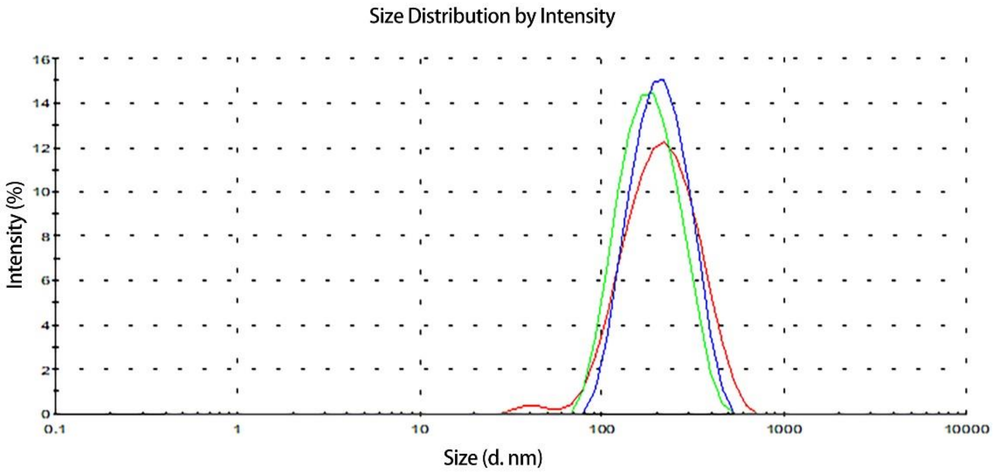

(b)

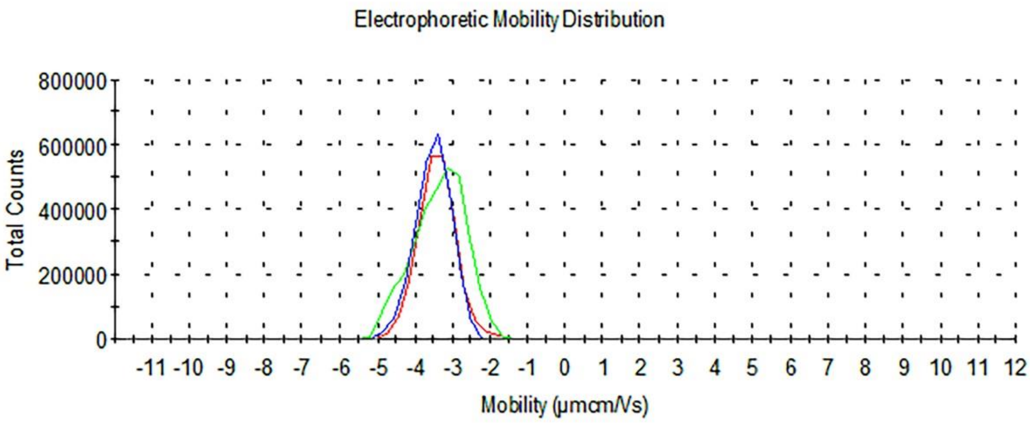

Figure S3. Size distribution (a) and zeta potential distribution (b) of krill PL liposomes

Table S1. Primers for qPCR assay

Genes	Forward primer sequence (5′-3′)	Reverse primer sequence (5′-3′)
iNOS	TCACGCTTGGGTCTTGTTCA	CCTTTTCCTCTTTCAGGTCACTT
COX-2	TGCACTATGGTTACAAAAGCTGG	TCAGGAAGCTCCTTATTTCCCTT
IL-6	TCCAGTTGCCTTCTTGGGAC	GTGTAATTAAGCCTCCGACTTG

Table S2. Calibration curves, linear regression coefficients (R^2), limit of detection (LOD) and limit of quantification (LOQ) of five PL standards

Standards	Formula	*m/z*	Equations	R^2	LOD (ng/mL)	LOQ (ng/mL)
15:0-18:1-d7-PE	$C_{38}H_{67}D_7NO_8P$	709.5519	$y = 1.94x+0.09$	0.9992	0.5	1.6
15:0-18:1-d7-PI	$C_{42}H_{72}D_7O_{13}P$	828.5625	$y = 3.28x-0.16$	0.9993	0.3	1.0
15:0-18:1-d7-PC	$C_{41}H_{73}D_7NO_8P$	811.6189	$y = 1.33x-0.14$	0.9996	0.6	2.0
15:0-18:1-d7-PA	$C_{36}H_{62}D_7O_8P$	666.5097	$y = 2.93x-1.15$	0.9989	0.4	1.3
15:0-18:1-d7-PG	$C_{39}H_{68}D_7O_{10}P$	740.5464	$y = 4.17x-0.12$	0.9991	0.3	1.0
